# Factors Associated with Congenital Heart Disease in Severely Malnourished Children under Five and Their Outcomes at an Urban Hospital, Bangladesh

**DOI:** 10.3390/children9010001

**Published:** 2021-12-21

**Authors:** Abu Sadat Mohammad Sayeem Bin Shahid, Tahmina Alam, Mst. Mahmuda Ackhter, Md. Zahidul Islam, Irin Parvin, Shamsun Nahar Shaima, Lubaba Shahrin, Tahmeed Ahmed, Fahmida Chowdhury, Mohammod Jobayer Chisti

**Affiliations:** 1 Nutrition and Clinical Services Division, International Centre for Diarrhoeal Disease Research, Dhaka 1212, Bangladesh; drtahmina@icddrb.org (T.A.); mahmuda.ackhter@icddrb.org (M.M.A.); zahidul.islam@icddrb.org (M.Z.I.); irin.parvin@icddrb.org (I.P.); shamsun.shaima@icddrb.org (S.N.S.); lubabashahrin@icddrb.org (L.S.); tahmeed@icddrb.org (T.A.); chisti@icddrb.org (M.J.C.); 2 Infectious Diseases Division, International Centre for Diarrhoeal Disease Research, Dhaka 1212, Bangladesh; fahmida_chow@icddrb.org

**Keywords:** congenital heart disease, malnutrition, children, outcome, Bangladesh

## Abstract

Congenital heart disease (CHD) is one of the most common types of birth defect with a high morbidity and mortality, particularly in severely malnourished children under five. In this study, we aim to identify the predicting factors for CHD and their outcomes. 694 malnourished children under five years of age admitted between April 2015 and December 2017 constituted the study population. Of them, 64 were cases of CHD, and by comparison 630 were without CHD. CHD was diagnosed clinically and confirmed by echocardiogram. 64% of the cases had a single defect. Cases were more likely to be present with diarrhea, cough, respiratory distress, cyanosis, hypoxemia, hypoglycemia and hypernatremia on admission. The cases also had a high proportion of severe sepsis, bacteremia, heart failure, respiratory failure and death, compared to those without CHD. Cough (95% CI = 1.09–18.92), respiratory distress (95% CI = 1.46–5.39) and hypoxemia (95% CI = 1.59–6.86) were found to be the independent predictors for CHD after regression analysis, and their early identification might be helpful to lessen ramifications, including mortality, in such populations, especially in resource-limited settings.

## 1. Introduction

Childhood malnutrition is a major public health concern worldwide [1]. Severe acute malnutrition (SAM) is one of the devastating conditions associated with high morbidity and mortality among children under five globally and its highest burden has been observed in developing countries [2,3,4]. Congenital heart disease (CHD) is the most frequently occurring congenital defect, affecting 0.8% of live births and leading to neonatal and infantile deaths, if untreated [5,6,7]. Thus, it is imperative to identify the predicting factors for CHD in children under five years of age with severe malnutrition in order to develop appropriate management guidelines to lessen ramifications, including mortality, especially in resource-limited settings.

The aim of our study was to recognize the predicting factors for CHD in such children and their outcomes, such as severe sepsis, bacteremia, heart failure, respiratory failure and death during hospitalization.

## 2. Materials and Methods

### 2.1. Study Site

The description of the study site has been provided elsewhere [8].

### 2.2. Study Population and Design

The description of the study population has been provided elsewhere [8]. Severe malnutrition has been defined following World Health Organization (WHO) reference [9].

Study profile has been described elsewhere [8]. We analyzed 64 children with CHD, which constituted both clinically and echocardiographically proven cases, and 630 children without CHD formed the comparisons.

### 2.3. Patient Management

All the enrolled children were managed following hospital’s standard protocol [10,11].

### 2.4. Measurements

Data on clinical and laboratory characteristics were collected and recorded on admission following standard procedure and case definition [12].

Outcome characteristics included severe sepsis [13], bacteremia [14], heart failure [15], respiratory failure [16] and death during hospitalization.

### 2.5. Statistical Analysis

Statistical analysis was carried out using standard procedures that have been provided elsewhere [12]. For investigating independent predicting factors for CHD in children with severe malnutrition, we first analyzed the relevant variables in a univariate model. We then adjusted the covariates by carrying out a regression analysis, where CHD was set as the dependent variable and the independent variables were those who were significantly associated with CHD in the bivariate analysis.

## 3. Results

Among 1163 children, 694 met our inclusion criteria. 64 (9%) of them had CHD and 630 (91%) did not. Clinical and laboratory characteristics were analyzed between the two groups of children (Table 1). Among the children with CHD, 64% and 36% had single and multiple defects, respectively.

**Table 1:** Characteristics of children between cases and comparisons at hospitalization.

Variables	Cases (*n* = 64)	Comparisons (*n* = 630)	OR	95% CI	*p*-Value
Male sex	41 (64)	386 (61)	1.12	0.66–1.92	0.660
Age in months	8.0 (5.0, 12.0)	10.0 (5.0, 16.0)	-	-	0.180
Working mother	4 (6)	83 (13)	0.43	0.15–1.23	0.100
Breastfeeding	48 (75)	448 (71)	1.19	0.66–2.15	0.560
Residence in slum	3 (5)	73 (12)	0.37	0.11–1.22	0.090
BCG vaccination	57 (89)	554 (88)	1.11	0.49–2.53	0.790
Diarrhea	48 (75)	560 (89)	0.37	0.20–0.69	0.001
Dehydration	7 (14)	104 (18)	0.74	0.32–1.71	0.490
Cough	61 (95)	295(47)	23.10	7.17–74.36	<0.001
Respiratory distress	46 (72)	128 (20)	10.02	5.62–17.87	<0.001
Lethargy	3 (5)	12 (2)	2.53	0.69–9.22	0.140
Convulsion	1 (2)	4 (1)	2.48	0.27–22.56	0.400
Cyanosis	3 (5)	1 (0.2)	30.93	3.17–301.9	<0.001
Pedal edema	1 (2)	52 (8)	0.17	0.02–1.29	0.054
Grunting	6 (9)	32 (5)	1.93	0.77–4.81	0.150
Hypoxemia	19 (30)	26 (4)	9.80	5.05–19.07	<0.001
Hypoglycemia	5 (8)	9 (1)	5.85	1.90–18.01	<0.001
Hypernatremia	9 (14)	44 (7)	2.17	1.01–4.70	0.040
Hyponatremia	4 (6)	334 (53)	0.06	0.02–0.16	<0.001
Hyperkalemia	15 (23)	347 (55)	0.25	0.13–0.45	<0.001
Hypokalemia	7 (11)	89 (14)	0.75	0.33–1.69	0.480
Radial pulse	142.4 ± 15.4	137.0 ± 9.4	-	2.79–8.02	<0.001
Respiratory rate	52.6 ± 10.5	42.8 ± 9.8	-	7.05–12.49	<0.001
Total WBC count Duration of hospitalization	13,570 (10,615, 18,420) 6.0 (4.0, 9.0)	14,110 (11,050, 18,720) 4.0 (3.0, 8.0)	- -	- -	0.843 0.001

OR, odds ratio; CI, confidence interval; WBC, white blood cell.

Children with CHD had a higher death rate compared to those without (11%, 3%, *p*-value = 0.001) (Table 2).

Regression showed independent association of cough, respiratory distress and hypoxemia with CHD (Table 3).

## 4. Discussion

We observed that cough, respiratory distress and hypoxemia were revealed as the independent predictors for CHD in our study children.

Our observation of the association of cough in severely malnourished children with CHD is quite explainable. As severely malnourished children usually suffer from recurrent infections such as pneumonia due to a lack of innate immunity [17,18], they had coughs which may be found in comorbidity, such as CHD.

Another important observation of the association of respiratory distress in the form of the increased work of breathing in severely malnourished children with CHD is quite explicable. An increased work of breathing is related to reduced oxygen volume and mechanical collapse in the lungs in children, potentially leading to respiratory failure, followed by death. In children with CHD, there are also additional factors that may increase the risk for respiratory compromise compared to a normal child. In left-to-right shunts, there is a chance of an increased flow of blood through pulmonary vasculatures, resulting in interstitial edema, which may lead to a further decrease in lung volume and a predilection for hypoxemia. All these factors jointly lead to an increased work of breathing in children with CHD, characterized by pulmonary overcirculation [19].

Another important finding is the independent association of hypoxemia in children with CHD. In right-to-left shunts, there is unrestricted communication between the left and right ventricles and obstruction to pulmonary outflow. In such lesions, children are prone to develop deteriorating hypoxemia, as they are potentially at risk for hypercyanotic episodes. In a child with reduced pulmonary blood flow, we often find hypoplastic lungs, which are unable to allow sufficient perfusion, leading to further hypoxemia in children with CHD [19,20].

We also observed a significantly high proportion of death in severely malnourished children with CHD, particularly due to bacteremia, which is similar to previous studies [10,20].

We also experienced a high development of severe sepsis in children with CHD. The mechanism for such development is well known [21,22].

In this study, we also observed that children with CHD had a high proportion of heart failure during hospitalization. Severe malnutrition may lead to heart failure [11], which is often associated with comorbidity, such as CHD. Ventricular dysfunction, volume or pressure overload may be the associated factors with CHD in malnourished children. Sometimes the causes of cardiomyopathy overlap with heart failure, which is caused by ventricular dysfunction leading to detrimental manifestations, such as respiratory distress in such populations [15,23]. Hyponatremia and hypokalemia are the most common electrolyte abnormalities in children with heart failure, and several mechanisms counteract to produce such imbalances [24].

In heart failure, retention of fluid and electrolytes occur [25] which may further reduce cardiac output and circulatory volume. These events increase the activity of the heart to fulfil the demand of full fluid distribution of the body, thus resulting in heart failure [24,26].

Hypokalemia is a major electrolyte disorder essential for normal cellular function [12]. It has been linked with serious life-threatening events, including brady arrhythmias and death, particularly in severely malnourished children with low levels of potassium [27,28]. The body tries to maintain itself within very marginal limits by regulating its internal environment [29]. In heart failure, evidence suggests that normal serum potassium is required to be maintained for the reduction in case-fatality rate. Severe forms of this disorder should preferably be corrected using potassium supplementation [30], and in severely malnourished children it has already been recommended by the WHO to reduce potential deaths [31]. However, there is a recommendation of routine potassium replacement in cases of heart failure, despite normal potassium levels [32].

Malnutrition is one of the major consequences of both cyanotic and acyanotic congenital heart diseases [33], which may be due to various causes, including reduced calorie intake, enhanced calorie demand or a combination of both [34,35,36,37]. Persistent hypoxemia was also found to be associated with anorexia, leading to malnutrition [34]. Pulmonary hypertension can also cause malnutrition in children with CHD [38].

Dietary insufficiency may be one of the potentiating factors for developing malnutrition in children with CHD. Profound hypoxemia, malabsorption and maldigestion are the pivotal players, behind cardiac cachexia [39,40]. Therefore, the study recommends the promotion of nutritional rehabilitation as part of a standard management protocol for severely malnourished children to bridle the unpleasant events of CHD [40].

Identifying the predicting factors for CHD in severely malnourished children was a major strength, as well as the study design, which kept statistical errors to a minimum. One of the limitations of the study was the smaller sample size.

## 5. Conclusions

Findings of the study suggested that cough, respiratory distress and hypoxemia were independent predictors for CHD in severely malnourished children under five. Death was even higher in such children with CHD than their counterpart. In parallel to the judicious uses of antibiotics for the management of severe malnutrition aligned with the WHO recommendation, as well as fluid management during hospitalization and nutritional rehabilitation for severe malnutrition, early identification of those simple clinical parameters might be helpful to lessen ramifications, including mortality, especially in resource-constrained areas.

## Figures and Tables

**Table 2:** Outcomes of hospitalized children between cases and comparisons.

Variables	Cases (*n* = 64)	Comparisons (*n* = 630)	OR	95% CI	*p*-Value
Severe sepsis Bacteremia	8 (13) 4 (6)	33 (5) 12 (2)	2.58 3.43	1.14–5.87 1.07–10.98	0.027 0.050
Heart failure	11 (17)	24 (4)	5.24	2.43–11.28	<0.001
Respiratory failure	5 (8)	17 (3)	3.06	1.09–8.58	0.044
Death	7 (11)	19 (3)	3.10	1.56–6.12	0.001

OR, odds ratio; CI, confidence interval.

**Table 3:** Regression model to explore the independent association in children with CHD.

Variables	OR	95% CI	*p*-Value
Diarrhea	1.36	0.66–2.82	0.400
Cough	4.53	1.09–18.92	0.038
Respiratory distress	2.80	1.46–5.39	0.002
Cyanosis	6.70	0.63–71.08	0.110
Hypoglycemia	2.49	0.75–8.29	0.136
Hypoxemia	3.30	1.59–6.86	0.001
Hypernatremia	0.68	0.27–1.68	0.401
Hyponatremia	0.30	0.08–1.03	0.056
Hyperkalemia	1.58	0.78–3.19	0.205
Radial pulse	0.99	0.97–1.02	0.580
Respiratory rate	1.01	0.97–1.05	0.740
Duration of hospitalization	1.02	0.97–1.07	0.406

## Data Availability

De-identified data will be available upon request from the research administration of icddr,b.
